# Emotional state in children after acute trauma in social disasters: psychological and psychiatric assistance

**DOI:** 10.1192/j.eurpsy.2026.11997

**Published:** 2026-07-31

**Authors:** Y. Sidneva, E. Lvova, S. Valiullina

**Affiliations:** 1 Neurorehabilitation, Clinical and Research Institute of Emergency Pediatric Surgery and Trauma; 2 Neuropsychiatry, N.N.Burdenko National Medical Research Institute of Neurosurgery, Moscow, Russian Federation

## Abstract

**Introduction:**

Acute severe trauma in social disasters is accompanied by multiple stress factors of varying etiology, severity, and duration, often overlapping. In the context of phased care, prolonged hospitalization, and multiple surgical interventions, given the complex nature of the trauma, children must overcome not only physical pain but also emotional pain. Questions arise regarding the need for psychological and psychiatric support: how much and when to include in the treatment and rehabilitation process.

**Objectives:**

To study the characteristics of emotional reactions in children with severe acute traumatic injuries after social disasters; To determine the tactics of psychological and psychiatric management in the early stages of treatment and rehabilitation.

**Methods:**

Children with traumatic injuries admitted to the Institute ofor treatment and rehabilitation in the early period: I - 25 children (9-17 y.o.) with spinal injury and amputation; II - 35 children (11-18 y.o.) with spinal injury outside of social problems. All patients were clinically examined by a psychiatrist and psychologist upon admission and over time, with the additional use of scales and questionnaires. Neuropharmacotherapy was provided using medications from various pharmacological groups, depending on the identified disorders and current rehabilitation goals. Psychological support for patients was using Gestalt correction methods.

**Results:**

I gr - 100% of cases were diagnosed with an acute stress reaction, characterized by a shock reaction; subsequent persistent symptoms of high anxiety, emotional lability, sleep disturbances, intrusive memories of the traumatic event, and affective-behavioral reactions. II - 48.6% had elevated anxiety and depressive symptoms of a reactive nature, without persistent signs of an acute stress reaction. An algorithm for joint psychological and psychiatric management of children was developed and tested. It was found that all children in Group 1 (100%) required simultaneous supervision by a psychiatrist and psychologist, specific medication therapy, and psychological correction. In the second group, psychological and psychiatric care was determined based on the severity of emotional disturbances: 48.6% of children required combined support with psychotherapy alongside neuropharmacotherapy.

**Conclusions:**

In the context of social disasters, children with traumatic injuries require mandatory joint psychological and psychiatric care, beginning from the early stages of treatment. Joint support (by a psychologist and psychiatrist) will allow for a timely, differentiated assessment of emotional disturbances and the determination of the most appropriate management strategy and neuropharmacotherapy for each patient, with the aim of increasing the effectiveness of treatment and rehabilitation and improving the child’s adaptive capacity in later life following traumatic events.

**Disclosure of Interest:**

None Declared

